# Effect of adjuvant therapy with teriparatide in patients with thoracolumbar osteoporotic vertebral fractures who underwent vertebroplasty with posterior spinal fusion

**DOI:** 10.1038/s41598-022-12655-x

**Published:** 2022-05-25

**Authors:** Yohei Shibuya, Keiichi Katsumi, Masayuki Ohashi, Hideki Tashi, Tatsuo Makino, Akiyoshi Yamazaki, Toru Hirano, Kimihiko Sawakami, Ren Kikuchi, Hiroyuki Kawashima, Kei Watanabe

**Affiliations:** 1 Department of Orthopedic Surgery, Niigata University School of Medicine, 1-757 Asahimachidori, Chuoku, Niigata City, Niigata 951-8510 Japan; 2 Spine Center, Department of Orthopedic Surgery, Niigata Central Hospital, Niigata, Niigata Japan; 3 Department of Orthopedic Surgery, Uonuma Institute of Community Medicine, Niigata University Medical and Dental Hospital, Minami-Uonuma, Niigata Japan; 4 Department of Orthopedic Surgery, Niigata City General Hospital, Niigata, Niigata Japan; 5 Department of Orthopedic Surgery, Niigata Rosai Hospital, Joetsu, Niigata Japan

**Keywords:** Diseases, Medical research

## Abstract

Teriparatide (TPTD) administration has a potent osteogenic action and promotes the healing of osteoporotic vertebral fractures (OVFs). We aimed to investigate the outcomes of vertebroplasty with posterior spinal fusion (VP + PSF) and determine the impact of perioperative TPTD administration. We included 73 patients (18 male and 55 female patients; mean age: 78 years) with thoracolumbar OVFs who underwent VP + PSF and were followed-up for at least 2 years. Twenty-three patients who received TPTD perioperatively for > 3 months were included in the TPTD group, and the remaining 50 patients were included in the non-TPTD group. Radiographic findings regarding sagittal alignment and clinical outcomes in both groups were compared. The mean duration of TPTD administration was 17.5 ± 5.0 months (range 4–24 months). The mean loss of correction of local kyphosis angle in the TPTD group (4.0°) was lesser than that in the non-TPTD group (7.5°; p < 0.05); however, no significant differences were observed between the groups regarding global sagittal alignment, the occurrence of subsequent vertebral fractures, pedicle screw loosening and treatment-efficacy rates of clinical outcomes. Local kyphosis correction in patients who underwent VP + PSF for OVFs could be maintained through perioperative TPTD administration; however, TPTD administration had little effect on clinical outcomes.

## Introduction

Thoracolumbar osteoporotic vertebral fractures (OVFs) are common fractures of the spine among older patients^1,2^; these fractures frequently cause neurological symptoms, including impairment of the spinal cord or cauda equina. Previous reports have shown that neurological deficits following OVFs are primarily caused by the instability of fracture sites rather than neural compression by retropulsed bone fragments^3–5^. Consequently, a large variety of surgical fusion techniques, including anterior spinal fusion, combined anterior and posterior spinal fusion, and posterior spinal fusion (PSF) with three-column osteotomy or vertebroplasty (VP), have been used to treat OVFs^6^. Among these techniques, VP with PSF (VP + PSF) is widely used to treat patients with OVFs who have intractable back pain or neurological symptoms because although this technique is associated with a limited ability for the correction of kyphosis, compared to other procedures, it is less invasive and is associated with a lower perioperative complication rate^4,6–9^. Additionally, high incidences of subsequent vertebral fractures after initial spinal fusion (ranging from 20 to 55%) have been reported in previous studies^3,4,6,7,10^. Regardless of the surgical method used, the occurrence of vertebral fractures is a common consequence of spinal-fusion surgery. Therefore, the implementation of preventive measures is crucial.

Teriparatide (TPTD), which is a recombinant form of human parathyroid hormone (containing the first 34 amino acids of the hormone) that acts directly on osteoblasts, has a potent osteogenic action from the early stages of its administration; the ability to increase bone mineral density (BMD) and reduce fracture incidence through the administration of TPTD has been reported previously^11–13^. With respect to fracture healing during conservative treatment, TPTD administration may enhance fracture healing and improve fracture union rates among patients with OVFs^14^. Recent studies have reported that adjuvant therapy with the administration of TPTD can result in a range of outcomes among patients who undergo spine surgeries, including enhancement of pedicle screw fixation^15,16^, prevention of subsequent vertebral fractures^17–19^, and improvement of spinal fusion rates^20,21^ due to its bone anabolic effect. However, although the efficacy of treatment with TPTD in patients with different degenerative disorders who were treated using different types of surgical methods has been analyzed in previous studies, there is a paucity of investigations focusing on specific fusion procedure.

In this study, we hypothesized that perioperative TPTD administration would have a positive effect on fusion rate, kyphosis correction, and the occurrence of subsequent vertebral fracture in patients with osteoporosis and thoracolumbar OVFs who underwent VP + PSF, which is the most versatile spinal fusion procedure. To test this hypothesis, we conducted a retrospective review of data from a multicenter database regarding patients with thoracolumbar OVFs to evaluate surgical outcomes and determine the impact of TPTD administration during perioperative periods.

## Results

TPTD administration was initiated > 3 months before surgery, ≤ 3 months before surgery, ≤ 1 week before or after surgery, and at the first outpatient-clinic appointment following discharge in 2, 6, 10, and 5 cases, respectively. The mean duration of TPTD administration was 17.5 months (standard deviation [SD]: 5 months; range 4–24 months); the mean duration of preoperative TPTD administration was 1.7 months (SD: 5.1 months), and that of postoperative TPTD administration was 15.7 months (SD: 5.9 months). There was only one case who received TPTD administration for less than 12 months.

### Radiological outcome

**Figure 1:**
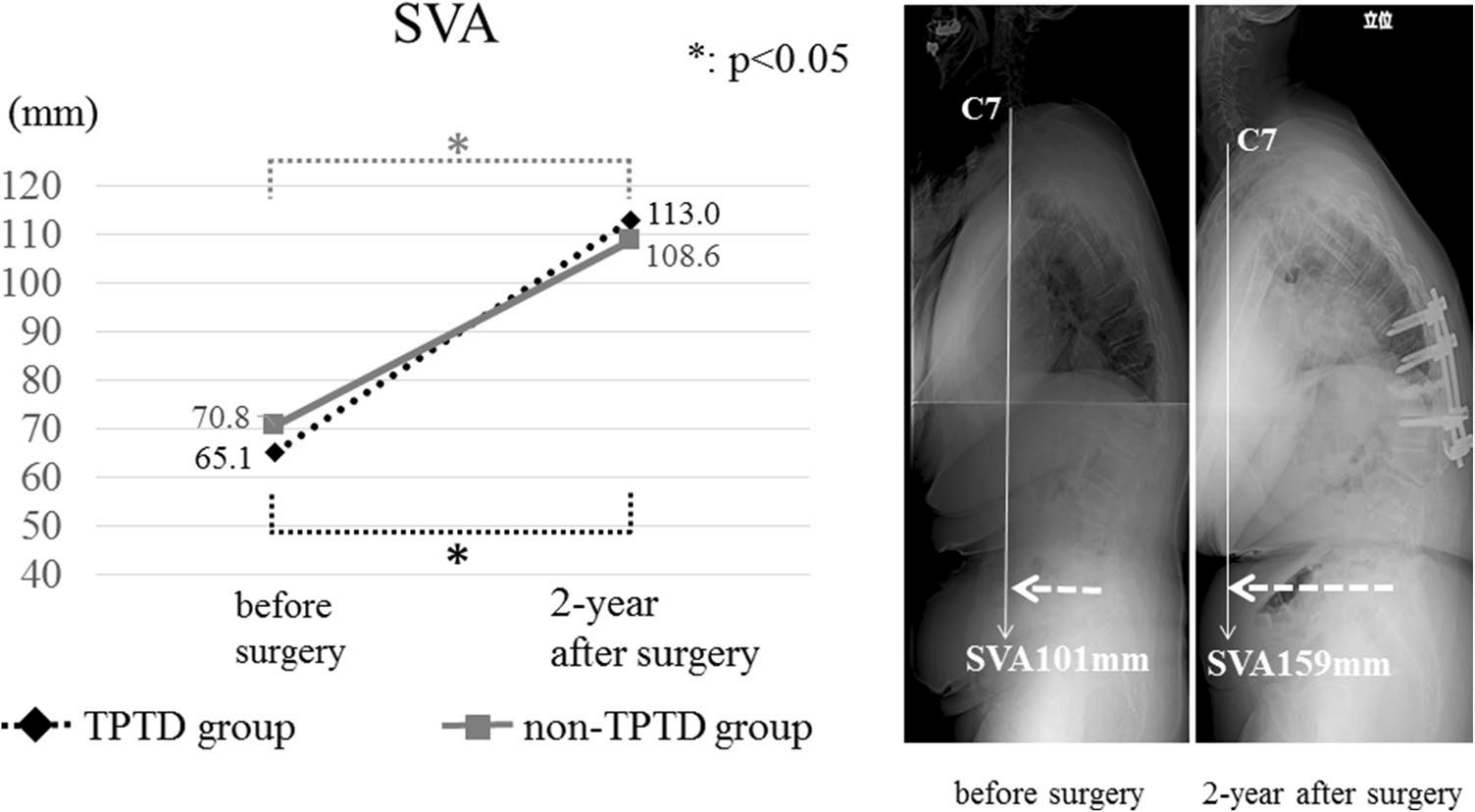
Mean sagittal vertical axis (SVA) observed preoperatively and 2 years after surgery; the figure shows that there was significant deterioration in both groups (*TPTD* teriparatide).

**Figure 2:**
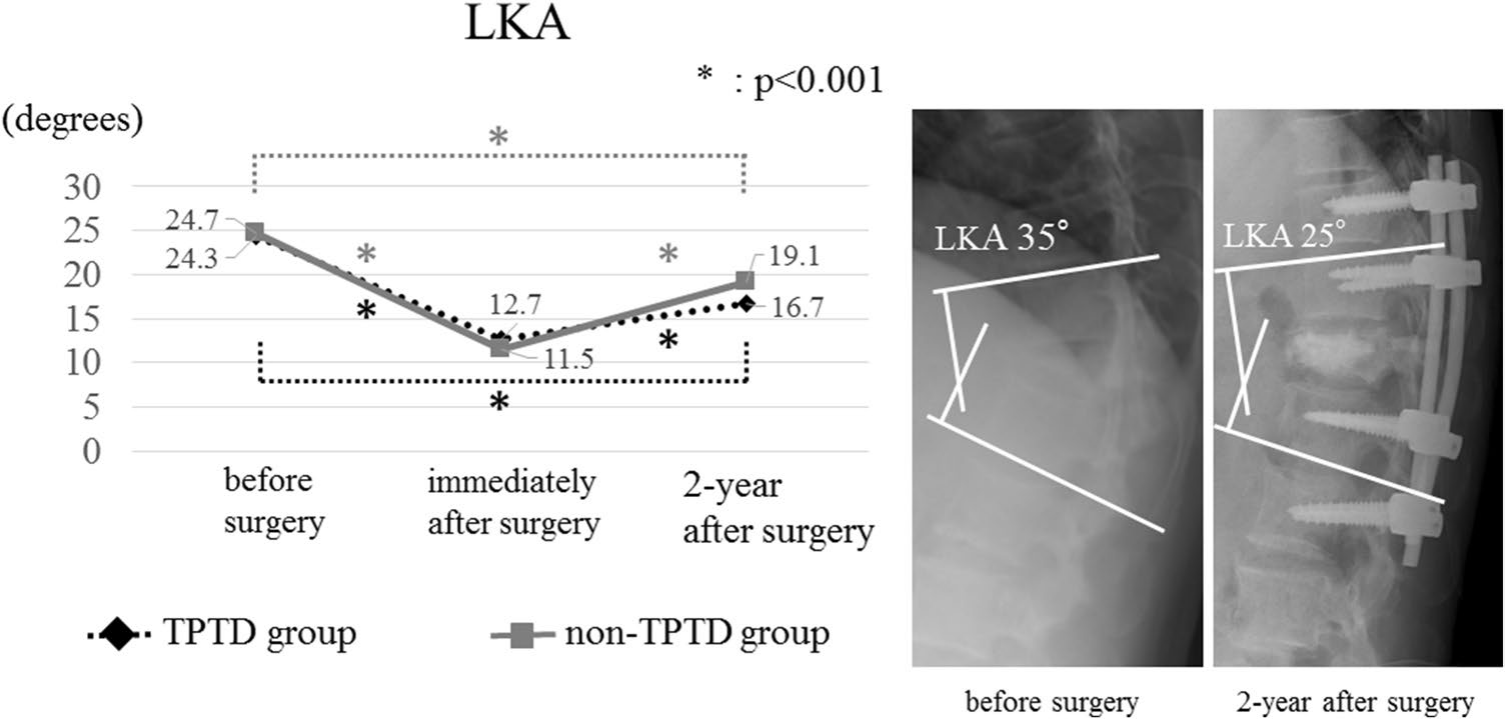
Mean local kyphosis angle (LKA) observed preoperatively, immediately after surgery, and 2 years after surgery; the figure depicts the changes in LKA and reveals that there was significant correction in both patient groups (*TPTD* teriparatide).

Demographic and radiological outcomes are presented in Tables 1 and 2. There were no significant differences between the two groups with respect to global sagittal alignment before and after surgery; however, sagittal alignment significantly deteriorated after surgery in both groups (Fig. 1). In the TPTD group, the mean local kyphosis angle (LKA) observed preoperatively, immediately after surgery, and 2 years after surgery were 24.3° (SD: 13.8°), 12.7° (SD: 11.0°), and 16.7° (SD: 11.4°), respectively; in the non-TPTD group, the mean LKA observed preoperatively, immediately after surgery, and 2 years after surgery were 24.7° (SD: 11.4°), 11.5° (SD: 10.0°), and 19.1° (SD: 9.4°), respectively. Although there was no significant difference between the two groups at any time, the mean loss of correction of LKA in the TPTD group was significantly lesser than that in the non-TPTD group (Fig. 2). There were no significant differences between the two groups with respect to the frequency of occurrence of subsequent fractures and pedicle screw loosening. Overall, 17 subsequent fractures (incidence: 23.3%) were identified, and 13 of the 17 fractured occurred within 3 months of surgery.

**Table 1:** Comparison of patient demographics.

	TPTD group	Non-TPTD group	p value
Number of patients (n)	23	50	
Age at surgery, mean ± SD (year)	77.1 ± 6.3	77.9 ± 5.8	0.64
Sex (man/woman)	5/18	13/37	0.78
**Fractured vertebra (n)**			0.99
Th10	1	2	
Th11	1	3	
Th12	11	26	
L1	7	14	
L2	3	5	
BMD of the femur, mean ± SD (g/cm^2^)	0.69 ± 0.12	0.70 ± 0.15	0.90
Existing vertebral fracture (Fx/no Fx)	14/9	26/24	0.48
Number of fused segments, mean ± SD (segment)	4.3 ± 1.3	3.7 ± 1.2	0.07
Follow-up period, mean ± SD (month)	37.8 ± 19.5	44.5 ± 23.4	0.27
**JOABPEQ, median (IQR: 25–75%)**			
Low back pain	42.9 (28.6–71.4)	28.6 (14.3–42.9)	0.06
Lumbar function	0.0 (0.0–66.7)	8.3 (0.0–45.8)	0.85
Walking ability	21.4 (14.3–50.0)	14.3 (0.0–32.1)	0.23
Social life function	25.7 (0.0–43.2)	14.9 (0.0–37.8)	0.74
Mental health	29.1 (13.6–58.3)	32.0 (16.0–41.3)	0.44

*SD* standard deviation, *IQR* interquartile range, *BMD* bone mineral density, *Fx* fracture, *JOABPEQ* Japanese Orthopaedic Association Back Pain Evaluation Questionnaire.

**Table 2:** Comparison of outcomes.

	TPTD group	Non-TPTD group	p value
**SVA, mean ± SD (mm)**			
Before surgery	65.1 ± 52.7	70.8 ± 42.6	0.49
2-year after surgery	113.3 ± 61.0	108.6 ± 67.2	0.85
**LKA, mean ± SD (°)**			
Before surgery	24.3 ± 13.8	24.7 ± 11.4	0.93
Immediately after surgery	12.7 ± 11.0	11.5 ± 10.0	0.78
2-year after surgery	16.7 ± 11.4	19.1 ± 9.4	0.36
Correction loss	4.0 ± 4.5	7.6 ± 5.9	0.02
Subsequent fracture (%)	30.4	24.0	0.58
Pedicle screw loosening (%)	34.8	48.0	0.32
Vertebral union rate (%)	100.0	98.0	0.99
**JOABPEQ effectiveness rate (%)**			
Low back pain	40.0	61.8	0.22
Lumbar function	68.8	51.4	0.36
Walking ability	43.8	48.6	0.77
Social life function	43.8	60.0	0.37
Mental health	43.8	51.4	0.76

*SD* standard deviation, *SVA* sagittal vertical axis, *LKA* local kyphosis angle, *JOABPEQ* Japanese Orthopaedic Association Back Pain Evaluation Questionnaire.

### Clinical outcomes

Clinical outcomes are shown in Table 2. There were no significant differences between the two groups with respect to any of the Japanese Orthopaedic Association Back Pain Evaluation Questionnaire (JOABPEQ) subscale scores determined preoperatively and 2 years after surgery; furthermore, there were no significant differences between the two groups with respect to effectiveness rates that were calculated using JOABPEQ subscale scores.

### Comparison after propensity score matching

Demographic data and outcomes after propensity score matching are shown in Tables 3 and 4, respectively. After propensity score matching, it was found that the loss of correction of LKA in the TPTD group was significantly lesser than that in the non-TPTD group. It was also determined that there were no significant differences between the groups with respect to sagittal vertical axis (SVA), the incidences of subsequent fractures and pedicle screw loosening, and effectiveness rates associated with all of the JOABPEQ subscales.

**Table 3:** Comparison of patient demographics after propensity score matching.

	TPTD group	Non-TPTD group	p value
Number of patients (n)	20	20	
Age at surgery, mean ± SD (year)	76.9 ± 6.4	75.7 ± 5.5	0.50
Sex (men/women)	5/15	8/12	0.50
**Fractured vertebra (n)**			0.99
Th10	1	1	
Th11	1	1	
Th12	8	9	
L1	7	7	
L2	3	2	
BMD of the femur, mean ± SD (g/cm^2^)	0.69 ± 0.12	0.69 ± 0.15	0.72
Existing vertebral fracture (Fx/no Fx)	11/9	9/11	0.75
Number of fused segments, mean ± SD (segment)	4.3 ± 1.2	4.2 ± 1.5	0.68
Follow-up period, mean ± SD (month)	36.5 ± 19.7	42.1 ± 19.9	0.52

*SD* standard deviation, *BMD* bone mineral density, *Fx* fracture.

**Table 4:** Comparison of outcomes after propensity score matching.

	TPTD group	Non-TPTD group	p value
**SVA, mean ± SD (mm)**			
Before surgery	66.2 ± 57.9	89.9 ± 31.6	0.24
2-year after surgery	112.9 ± 64.7	113.1 ± 54.0	0.58
**LKA, mean ± SD (°)**			
Before surgery	23.8 ± 13.1	26.9 ± 11.9	0.53
Immediately after surgery	12.2 ± 10.0	13.3 ± 10.3	0.70
2-year after surgery	16.2 ± 11.3	21.1 ± 9.3	0.19
Correction loss	4.0 ± 4.6	7.9 ± 6.2	0.04
Subsequent fracture (%)	35.0	20.0	0.48
Pedicle screw loosening (%)	40.0	45.0	0.99
Vertebral union rate (%)	100.0	95.0	0.99
JOABPEQ effectiveness rate (%)			
Low back pain	40.0	41.7	0.99
Lumbar function	68.9	41.7	0.25
Walking ability	43.8	50.0	0.99
Social life function	43.8	50.0	0.99
Mental health	43.8	58.3	0.70

*SD* standard deviation, *SVA* sagittal vertical axis, *LKA* local kyphosis angle, *JOABPEQ* Japanese Orthopaedic Association Back Pain Evaluation Questionnaire.

## Discussion

In the present study, we evaluated the surgical outcomes of VP + PSF for thoracolumbar OVFs and determined the effect of TPTD administration during perioperative periods. We hypothesized that perioperative TPTD administration would have a positive effect on prevention of postoperative complications. Consequently, we found that perioperative TPTD administration was associated with a decrease in the loss of correction of LKA; however, it had little effect on clinical outcomes, including correction of global spinal alignment, prevention of the occurrence of subsequent vertebral fractures or pedicle screw loosening, and enhancement of quality of life.

Through the performance of VP + PSF, which is less invasive than other procedures, a significant improvement in back pain or neurological statuses can be achieved in patients; however, the technique is limited with respect to the correction of kyphosis^4,6–9^. The findings of the present study show that although global spinal alignment could not be improved through VP + PSF, local kyphosis correction could be maintained through TPTD administration. Inoue et al.^15^ reported that preoperative TPTD administration that is initiated at least 1 month prior to surgery increases the insertional torque of pedicle screws (TPTD group: 1.28 ± 0.42 Nm; control group: 1.08 ± 0.52 Nm). It has been reported in previous studies that the biomechanical pullout strength of pedicle-screw fixation is directly proportional to the torque at the time of screw insertion^22,23^. Ohtori et al.^20,24^ reported that preoperative TPTD administration prevented pedicle screw loosening and enhanced the rate of bone union after posterolateral lumbar fusion. Therefore, among the TPTD-group patients included in the present study, preoperative TPTD administration may have caused an improvement in the initial stability of posterior instrumentation, which could have resulted in the decreased loss of correction of LKA.

With respect to the occurrence of subsequent fracture after VP + PSF for osteoporotic OVFs, the rate of subsequent fracture previously reported by Katsumi et al.^4^ and Kashii et al.^7^ was 44% and 38%, respectively. Maruo et al.^19^ reported that preoperative and postoperative TPTD administration significantly reduced the incidence of subsequent vertebral fractures after instrumented fusion surgery for OVFs (TPTD group: 16%; non-TPTD group: 54%). It has also been reported that preoperative and postoperative TPTD administration causes a decrease in the occurrence of adjacent vertebral fractures following long instrumented fusion for adult spinal deformity^18^. However, among the patients included in the present study, 76% of subsequent vertebral fractures occurred within 3 months of surgery, and there was no significant difference between the two groups with respect to the incidence of subsequent vertebral fractures during perioperative periods (TPTD group: 30.4%; non-TPTD group: 24%). Previously, based on results associated with bone-formation markers and bone histomorphometry, it was postulated that the osteogenic response to TPTD treatment peaks after 6 to 12 months of TPTD administration and declines thereafter^25,26^. Through bone histomorphometry, Sawakami et al.^27^ found that osteogenic-parameter values reached their peaks 3 to 4 months after TPTD administration. In the present study, the durations of preoperative administration of TPTD in 21 of the 23 patients who received TPTD were ≤ 3 months; such durations may not be sufficient for the provision of a substantial anabolic effect. Therefore, early TPTD administration and preoperative TPTD-administration durations that are at least 3 months long may be considered to prevent the occurrence of subsequent vertebral fractures after VP + PSF.

This study has some limitations. There is some institutional bias associated with surgical indications, procedures, and criteria for the use of TPTD. Nevertheless, no significant differences were observed between the two groups with respect to age, sex, BMD of the proximal femur, fractured vertebrae, number of fused segments, and preoperative LKA. Another limitation is the relatively small number of patients included in the current study to whom TPTD had been administered. Propensity score matching was used to improve the accuracy of comparisons; however, there were no changes in the conclusions after propensity score matching. We believe that our conclusions are based on a relatively large number of patients who were treated using a uniform procedure, and it is reasonable to compare these two patient groups in a retrospective study. A further limitation of this study is that the duration of preoperative TPTD administration was short because it was often difficult to ensure an adequate period of conservative treatment when back or neurological symptoms were severe and interfered with patients’ daily lives. Therefore, it is our next assignment to examine the efficacy of TPTD adjuvant therapy on further improvement of clinical outcomes in a larger sample size with an increased number of patients with longer duration of preoperative administration. Although the effectiveness of TPTD administration in patients with OVFs has been recognized in recent years, evidence regarding its clinical effects in various clinical settings must be obtained. Therefore, further prospective studies are needed to determine the appropriate criteria for the use of TPTD or the timing of perioperative TPTD administration among patients who undergo spinal reconstructive surgery.

In this study, we have reported the clinical outcomes of VP + PSF and concomitant perioperative TPTD administration (for a mean duration of 17.5 months [mean preoperative administration duration: 1.7 months]) in patients with thoracolumbar OVFs. Perioperative TPTD administration caused a decrease in the loss of correction of LKA; however, it had little effect on clinical outcomes such as the correction of global sagittal alignment, prevention of subsequent vertebral fractures, and enhancement of quality of life.

## Methods

This study was approved by the ethics committee of Niigata university (approval number: 2015-1385). The study protocol was in accordance with the 1964 Helsinki declaration and its later amendments or comparable ethical standards, and informed consent was waived by the ethics committee of Niigata University School of Medicine owing to the study design. In this retrospective study, patients with OVFs who underwent surgical intervention between 2008 and 2018 at our university hospital and three of its affiliated hospitals were enrolled. Patients who had OVFs in the thoracolumbar junction (which extends from the T10 vertebra to the L2 vertebra), those who underwent VP with instrumented PSF, and those who were followed up for a minimum 2 years were included. Patients with an acute vertebral fracture due to high-energy injury or a pathological fracture were excluded from this study. Patients who had previously undergone thoracolumbar fusion surgery were also excluded. A total of 73 patients, including 18 male and 55 female patients, with a mean age at surgery of 77.6 years (range 63–89 years), were included in the analysis. The mean disease duration was 5.4 months (range 1–20 months); fresh fracture (range 1–3 months), delayed-union (range 3–9 months) and non-union (range 9–20 months) were in 17 cases, 45 and 11, respectively. The patients were divided into two groups depending on whether TPTD was administered during perioperative periods. Twenty-three patients to whom TPTD had been administered for more than 3 months were included in a TPTD group, and the remaining 50 patients were included in a non-TPTD group. In the non-TPTD group, patients treated by bisphosphonate, active vitamin D3, no medication were in 23 cases, 3, and 24, respectively. Although there were no significant differences in patient characteristics and baseline data, the number of fused segments and the pain-related subscale scores among the TPTD-group patients (as determined using the JOABPEQ) tended to be higher than those among the non-TPTD-group patients (Table 1).

### Surgical procedure

The basic indications for VP + PSF included intractable back pain (regardless of the presence of neurological symptoms) and neurological symptoms in patients in whom spinal canal occupation by retropulsed bone fragments was < 60%. The surgical procedure involves PSF, in which pedicle screws and a rod system are used, and VP, which is performed using hydroxyapatite blocks or the injection of bone cement via the transpedicular approach. Posterior autologous bone grafts were added to all segments of the instrumentation in all patients. All patients underwent in situ fixation while they were positioned on Relton-Hall frames, and we did not attempt to correct kyphosis using spinal-instrumentation techniques, such as rod cantilever techniques. Neural decompression was performed in 14 patients. Postoperatively, plastic thoracolumbar orthoses were used by the patients for 3–6 months.

### Evaluation

The timing and duration of TPTD administration, radiological outcomes, and clinical outcomes were evaluated using medical charts, plain standing radiographs, and computed tomography (CT) images. Parameters evaluated through radiological analysis included SVA, LKA, and the occurrence of subsequent vertebral fractures and pedicle screw loosening. In each patient, the LKA was measured by evaluating lateral radiographs using the Cobb method; the angle between the upper endplate of the uninvolved vertebra above the fractured level and the lower endplate of the uninvolved vertebra below the fractured level was considered the LKA (Fig. 2). For each patient, we calculated the angular loss of correction by subtracting the value of the LKA immediately after surgery from the value of the LKA 2 years postoperatively. A patient was considered to have pedicle screw loosening if for that patient, a lucent zone around a pedicle screw was observed through CT. A patient was considered to have vertebral union if continuous trabeculae in the patient were observed through CT and a range of motion of ≤ 2° was observed on lateral flexion–extension radiographs. Clinical outcomes were evaluated using the JOABPEQ; patients were given scores ranging from 0 to 100 points, with higher scores indicating better conditions^28^. Currently, a treatment method is considered effective for a particular patient if the postoperative score for the patient is at least 20 points greater than the patient’s preoperative score, or if the preoperative score is < 90 points and the postoperative score is ≥ 90 points. The rate of treatment effectiveness in each group was calculated using the following formula: (number of patients in the group for whom treatment was considered effective)/[(total number of patients in the group) − (number of patients whose preoperative and postoperative scores were ≥ 90 points)]^28^.

### Statistical analysis

Statistical analyses were performed using the SPSS software (version 19.0; IBM, Armonk, NY, USA). Regarding clinical and radiological outcomes, change from baseline was evaluated using the Wilcoxon rank-sum test. Differences between the two groups were evaluated using the Mann–Whitney U test for continuous variables and the χ^2^ test for categorical variables. Statistical significance was set at p < 0.05.

Additionally, we matched background data using propensity score matching. Propensity scores were initially calculated by considering the following variables: patient age and sex, BMD of the proximal femur, levels of fractured vertebrae, number of fused segments, and preoperative LKA. Calculations were conducted using a logistic regression model. The C-statistic suggested that the fit was 0.70, which is a fairly good score. The TPTD and non-TPTD groups were matched based on propensity scores, with the condition that caliper widths should be less than 0.1. Twenty pairs of patients in the TPTD and non-TPTD groups were created after matching, and clinical outcomes among the matched patients were then compared.

## Data Availability

Data can be available upon request.
